# Urinary bladder paraganglioma in pregnancy: a case report and literature review

**DOI:** 10.3389/fmed.2025.1527778

**Published:** 2025-08-14

**Authors:** Yadong Sun, Lili Chen, Yongxiang Li, Liang Qiao

**Affiliations:** ^1^Department of Urology, Weifang People’s Hospital, The First Clinical Medical College of Shandong Second Medical University, Weifang, China; ^2^Shangdong Provincial Key Laboratory for Prevention and Treatment of Urological Diseases in Medicine and Health, Weifang, China; ^3^Department of Thoracic Surgery, Union Hospital, Tongji Medical College, Huazhong University of Science and Technology, Wuhan, China

**Keywords:** urinary bladder tumor, pregnancy, paraganglioma, hypertension, rare disease

## Abstract

Urinary bladder paragangliomas (UBPs) are rare tumors of chromaffin tissue originating from the sympathetic innervation of the bladder wall. It constitutes less than 0.05% of bladder tumors and 0.7% of all paragangliomas. The occurrence of UBP during pregnancy is extremely rare. Hypertensive disorders of pregnancy due to paraganglioma significantly increase the risk of adverse maternal and fetal outcomes. Herein, we report the case of UBP in a 28-year-old woman at 34 weeks’ gestation. The baby was successfully delivered, and bladder tumor was excised via partial cystectomy during the same surgical procedure. In addition, we conducted a literature review of paragangliomas during pregnancy, as there are only a few case reports of such diseases and there is no consensus on standard treatment. This study aims to contribute to the enhancement of the diagnosis and treatment of UBP during pregnancy.

## 1 Introduction

Paragangliomas (PGLs) are rare tumors that typically secrete catecholamines, leading to hypertensive crises that can be fatal (1). One-third to one-half of them are located in the thoracoabdominal region (2, 3). Urinary bladder paraganglioma (UBP), a subtype of PGL originating from the paraganglia within the bladder wall, is among the rarest forms of PGLs, accounting for approximately 0.7% of all PGLs and less than 0.05% of all bladder tumors (4, 5). Besides, PGLs in pregnancy are rare. Estimates of the incidence of PGL in pregnancy vary widely, ranging from 1/15,000 to 1/300,000 pregnancies (6). Although PGLs rarely occur during pregnancy, if left unrecognized, it can lead to a high risk of maternal and/or fetal death. The extremely low prevalence of UBP presents a significant challenge in accurately diagnosing them as the cause of hypertension in pregnancy. When UBP is misdiagnosed as pre-eclampsia, the maternal mortality rate can be as high as 15% (7).

Scarce data are available on the management and outcomes of patients with UBPs. In addition, there is no consensus on the best therapeutic strategy to address this condition. To the best of our knowledge, only seven relevant case reports were retrieved from the English and Chinese literature. Herein, we report the case of a 28-year-old woman with UBP, in which the infant was successfully delivered and the tumor was excised at the same time. In addition, we have conducted a comprehensive literature review regarding PGL and UBP. Our goal is to enhance clinicians’ awareness of UBPs and provide a comprehensive diagnostic and management framework for this rare condition.

## 2 Case presentation

**Figure 1:**
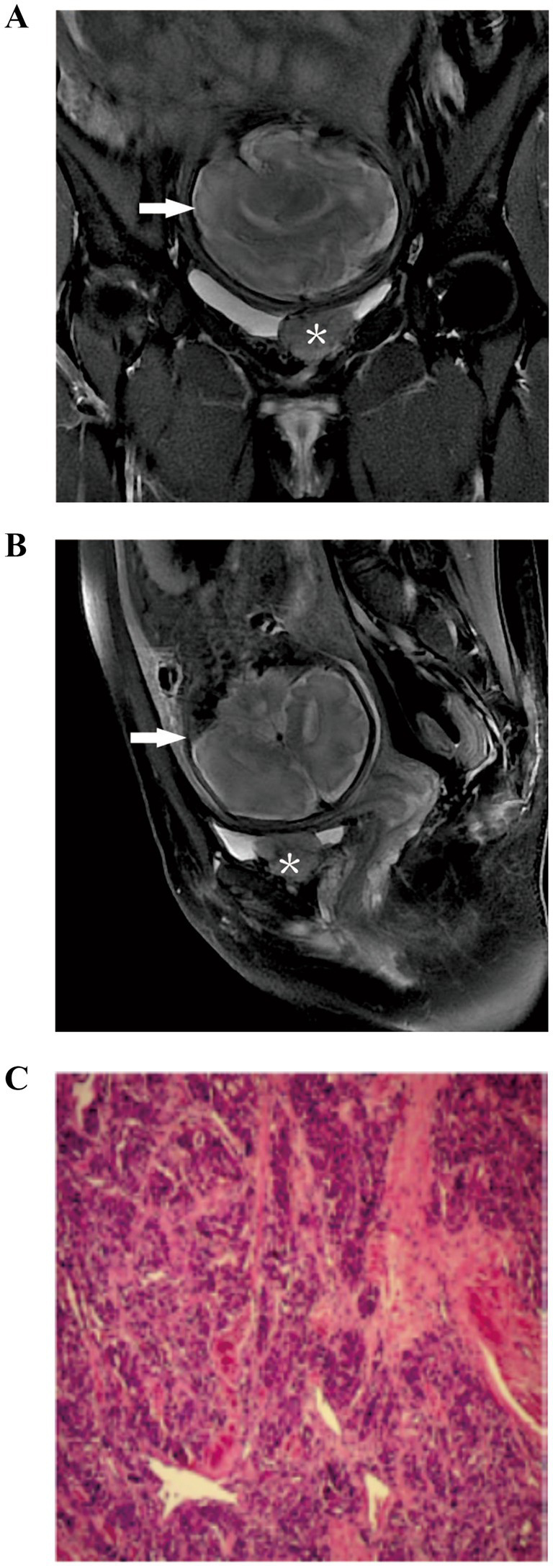
**(A)** Coronal and **(B)** sagittal fat saturation T2 weighted MRI demonstrates an infant (arrow) and a bladder tumor (asterisk). **(C)** Histological image of the UBP.

**Figure 2:**
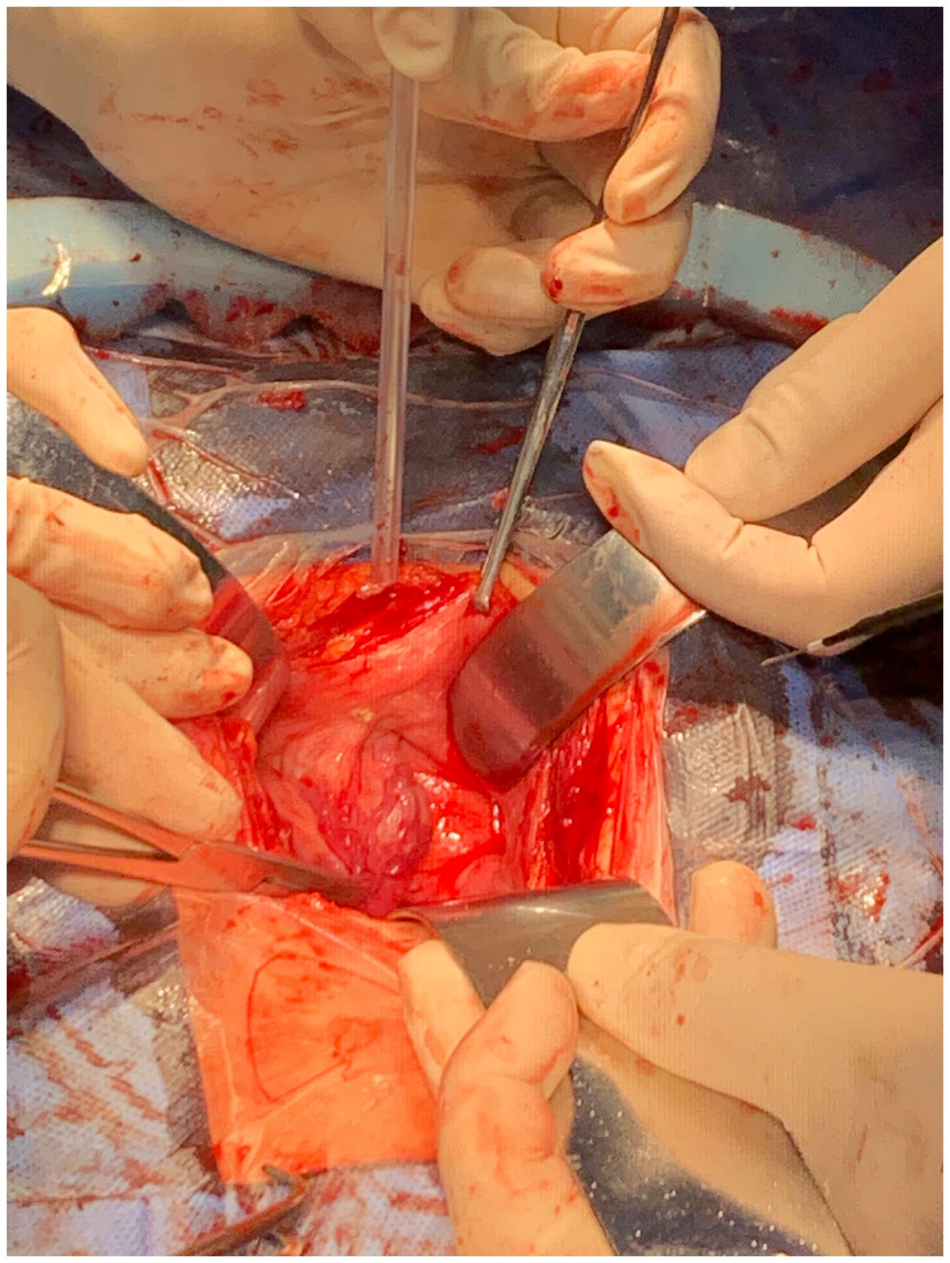
Intraoperative picture.

We present the case of a 28-year-old woman at 34 weeks’ gestation who presented to the obstetrics department with a 7-week history of hypertension, accompanied by headache and nausea. She had no history of hematuria and an urgent and frequent need of urination, with no known drug or food allergies. The patient is a non-smoker and non-alcoholic, working as a middle-school teacher, with no family history of hypertension and urinary bladder carcinoma. Her blood pressure (BP) remained normal in the current pregnancy after taking labetalol hydrochloride (100 mg Q8h) and phenoxybenzamine (10 mg Q12h). Plasma normetanephrine showed an exceedingly elevated level of 4.387 umol/l (normal range 0–0.6). Magnetic resonance imaging (MRI) of the abdomen and pelvis revealed a viable fetus (Figures 1A,B, arrows) and a highly vascular bladder mass measuring 4.0 × 3.0 × 2.1 cm (Figures 1A,B, asterisks), covering the left lateral wall of the urinary bladder. The left ureteric orifice was indistinguishable from the bladder tumor. Due to the high risk of pre-eclampsia, cystoscopy was not performed. A multidisciplinary team, comprising urology, obstetrics, endocrinology and anesthesia, decided to perform a cesarean section, followed by a partial cystectomy. Before surgery, physical examination findings were as follows: temperature, 36.8°C; pulse, 80 beats/min; respiration, 20 beats/min; and blood pressure, 130/80 mmHg. The entire operation lasted 2 h and 9 min, with an estimated blood loss of 30 mL. The intraoperative picture is shown in Figure 2. The baby, who had Apgar scores of 10 and 10 at both 1 and 5 min, respectively, was admitted to the neonatal unit for observation. As shown in Figure 1C, the histological image of the tumor shows epithelioid chief cells arranged in nests, separated by prominent fibrovascular stroma. The immunohistochemistry results confirmed that the tumor was a paraganglioma with Vimentin (+), CD56 (+), Syn (+), CgA (+), inhibin-*α* (+), GATA3 (+), Ki-67 (index 2%) and S-100 (+). The pathologist found no signs of vascular or neural invasion of the tumor. A blood test on the first postoperative day revealed a hemoglobin level of 92 g/L, representing a 19 g/L decrease from the preoperative value, while other indicators were similar to the preoperative level. Anti-hypertensive medications were stopped on postoperative day 2, and the mother’s BP remained normal. She was discharged from the hospital 7 days after the operation and was regularly followed up for 10 months after the operation, with no signs of hypertensive and tumor recurrence.

## 3 Discussion

Paraganglioma (PGL) is a neuroendocrine neoplasm, occurring at almost any body location, except within the brain and bones (8). All tumors have the potential to metastasize. They are referred to as metastatic or non-metastatic tumors, rather than being labeled benign or malignant. A unique feature of PGL is its capacity to synthesize and secrete catecholamines. Urinary bladder paraganglioma (UBP), accounting for approximately 0.7% of all PGLs, is one of the rarest types of PGLs. Current experiences of diagnosis and treatment of UBPs are based on that of PGLs. The diagnosis of UBP relays on biochemical tests, such as 24-h urine measurements of catecholamines or metabolites. Besides, it can be diagnosed through clinical symptoms, such as headache, hypertension, and perspiration, which were present in nearly 80% of patients (9). Imaging methods, such as ultrasound and MRI, are primarily used to access tumor location and volume. On MRI, a lobulated, oval, well-defined lesion may be found in all walls of the urinary bladder. On T1-weighted imaging, the signal intensity is similar to that of a muscle. Increased signal can be detected on T2-weighted imaging, with well-defined flow voids (10). Differential diagnosis of UBP is difficult in the absence of typical symptoms and negative biochemical tests. In this case, the differential diagnoses include the following: (1) urinary bladder carcinoma, which is the most common bladder tumor, and this case does not have the typical symptoms of UBP, and (2) bladder endometriosis, which is the most frequent type of urinary tract endometriosis, occur in 70–85% of cases. It is associated with lower urinary tract symptoms such as hematuria, frequency, and urgency (11). We performed urinary cytology to help differentiate bladder carcinoma from others. The examination of voided urine or bladder-washing specimens for exfoliated cancer cells has high sensitivity in high-grade (HG) tumors (84%) but low sensitivity in low-grade (LG) tumors (16%) (12). Negative results, although it is not certainly accurate, suggested that the tumor is not bladder cancer. Given the possibility of pre-eclampsia, the Multi-Disciplinary Treatment (MDT) panel did not recommend biopsy or diagnostic transurethral resection of bladder tumors (TURBT).

The other barrier to the diagnosis of UBP in pregnancy is distinguishing it from the more common diagnoses of gestational hypertension and/or pre-eclampsia (13). Gestational hypertension and pre-eclampsia are diagnosed as developed hypertension after a gestation period of 20 weeks (14). In contrast, the hypertension caused by UBP can be developed before 20 weeks of gestation. Moreover, UBP-related hypertension is generally not accompanied by ankle edema or elevated plasma uric acid levels, which would otherwise be common clinical manifestations of gestational hypertension or pre-eclampsia (15). Once the UBP is provoked, such as by holding urine or performing an intravesical operation, the patient’s blood pressure is elevated to a greater extent. Therefore, we did not perform a diagnostic transurethral resection of bladder tumor (TURBT) before surgery to take biopsies for pathological diagnosis to clarify the nature of the tumor. However, Dattatrya et al. reported that a woman at 9 weeks and 2 days of gestation with a UBP measuring 6.5- x 5.5 cm safely underwent a TUR biopsy (16). In addition, Song et al. also reported a safe cystoscopy on a 31-year-old woman with 8-cm UBP at 36 weeks of gestation (17). The entire procedure was uneventful. This case suggests that TUR biopsy might be a safe option during both early and late stages of pregnancy.

Since there are no standard procedures to diagnose UBP, we searched PubMed for reports on UPB with pregnancy from 1968 to the present. Only 7 case reports were found, and none of them provided high-resolution magnetic resonance images. Among these reports, Bakri et al. reported a misdiagnosis of UBP (18), where a 40-year-old woman was referred to the outpatient clinic due to a molar pregnancy. Preoperatively misdiagnosed as an invasive trophoblastic tumor, the UBP was resected during a planned abdominal hysterectomy for molar pregnancy. The diagnosis was confirmed only after microscopic study of the resected bladder tumor. In addition, Bassoon-Zaltzman et al. reported a case of a woman at 20 weeks of gestation, with episodes of non-sustained ventricular tachycardia, which were controlled with amiodarone (19). The UBP had not been diagnosed during the pregnancy. She had undertaken the 24-h urine catecholamine levels test. The catecholamine levels were markedly elevated. However, neither computed tomography nor postpartum metaiodobenzylguanidine scan could locate the tumor. Finally, the UBP was identified by ultrasound after selective venous sampling localized it to the pelvis. In the other five cases, the UBPs were preoperatively diagnosed successfully, either by ultrasound or biochemical tests. The biochemical tests mainly refer to the elevated levels of fractionated metanephrines and catecholamines in plasma and urine. UBP should be strongly considered when values are 3–4 times higher than the upper reference limit (20). Besides, unusual preoperative features rarely exist, constituting a diagnostic challenge to urologists (21, 22). Based on our case and the above cases taken together, we suggest that the diagnosis of UBP during pregnancy should be adequately combined with clinical symptoms, biochemical tests, and MRI imaging.

Once the UBP is diagnosed properly, appropriate treatment should be initiated promptly. Based on the treatment approach for UBP in non-pregnant patients, preoperative medical control of BP and subsequent surgical removal remains the definitive treatment (23). Among the seven pregnancy cases retrieved from the literature, except for the one case in which the UBP was not diagnosed during pregnancy, all six other cases underwent concurrent tumor resection along with cesarean section without adverse maternal or fetal consequences. There is currently no evidence to suggest which surgical procedure is superior for the removal of UBP. All cases were given *α*-blockade before surgery. Phenoxybenzamine, a selective α-blockade, is considered safe during pregnancy (24). BP should be medically controlled for a minimum of 10 days before surgery (25). En bloc resection has recently emerged as a treatment option for bladder cancer, especially for tumors smaller than 3 cm in size (28, 29). It may also be considered a promising approach for managing UBP.

PGLs, included UBPs, are among the most highly heritable human tumors, with 30–40% of cases being associated with germline pathogenic variants (GPVs) in up to 16 genes (1). Bancos et al. found that 66% of patients with PGL during pregnancy who underwent genetic testing had a GPV, including 19% in SDHB, 19% in RET and 13% in VHL (26). We have also previously reported that bladder carcinoma gene mutations affect treatment outcomes (27). Because of the high rate of hereditary diagnosis, all patients with gestational UBP should undergo genetic counseling and testing, preferably on a multigene panel that includes at least Succinate Dehydrogenase Complex Iron Sulfur Subunit B (SDHB), Ret Proto-Oncogene (RET), Von Hippel-Lindau Tumor Suppressor (VHL), Succinate Dehydrogenase Complex Iron Sulfur Subunit A (SDHA), Succinate Dehydrogenase Complex Iron Sulfur Subunit C (SDHC), and Succinate Dehydrogenase Complex Iron Sulfur Subunit D (SDHD). A positive result can have important implications for the patient, her children, and other first-degree relatives (30).

## 4 Conclusion

UBP in pregnancy is extremely rare. The diagnosis of UBP during pregnancy should be adequately combined with clinical symptoms, biochemical tests, and MRI imaging. Once UBP is diagnosed in pregnancy after the second trimester, concurrent cesarean section and partial cystectomy is safe as long as preoperative preparation is adequate, although strict intraoperative blood pressure monitoring is still required. From the high rate of hereditary diagnosis, all patients with gestational UBP should be offered genetic counseling and testing.

## Data Availability

The raw data supporting the conclusions of this article will be made available by the authors, without undue reservation.
